# Predictive value of Geriatric Nutritional Risk Index for overall survival in maintenance hemodialysis patients

**DOI:** 10.3389/fnut.2025.1640061

**Published:** 2025-10-08

**Authors:** He He, Yanlang Yang

**Affiliations:** ^1^Department of Nephrology, The Second Affiliated Hospital of Wannan Medical College, Wuhu, Anhui, China; ^2^Department of Nephrology, The First Affiliated Hospital of Wannan Medical College, Wuhu, Anhui, China

**Keywords:** Geriatric Nutritional Risk Index (GNRI), maintenance hemodialysis (MHD), overall survival, nutritional status, prognostic value

## Abstract

**Objective:**

This study aims to assess the predictive value of the Geriatric Nutritional Risk Index (GNRI) for overall survival in maintenance hemodialysis (MHD) patients, providing insights into the role of nutritional status in long-term outcomes for this patient population.

**Methods:**

A retrospective cohort study was conducted using data from 395 MHD patients treated between 2020 and 2024. The GNRI was calculated for each patient based on their baseline clinical data, including serum albumin, body mass index (BMI), and other relevant parameters. Patients were categorized into different groups according to their GNRI scores. Kaplan–Meier survival analysis was used to compare survival rates among different GNRI groups. Cox proportional hazards regression models were employed to evaluate the association between GNRI and overall survival, adjusting for potential confounding factors such as age, comorbidities, and other clinical indicators. The predictive accuracy of GNRI was further assessed using receiver operating characteristic (ROC) curve analysis.

**Results:**

The study found that patients with lower GNRI scores had significantly lower overall survival rates compared to those with higher scores. Specifically, patients in the lowest GNRI quartile had a higher mortality risk than those in the highest quartile. The Cox regression analysis showed that GNRI was an independent predictor of overall survival in MHD patients, with a hazard ratio (HR) indicating a substantial increase in mortality risk for each unit decrease in GNRI. The ROC curve analysis demonstrated that GNRI had a good predictive ability for overall survival, with an area under the curve (AUC) of 0.754 (95% CI: 0.7085–0.8064) suggesting its potential as a useful prognostic tool. At the optimal cut-off value of 93, the sensitivity was 78.2% and the specificity was 68.5%.

**Conclusion:**

The Geriatric Nutritional Risk Index (GNRI) is a valuable and reliable predictor of overall survival in maintenance hemodialysis patients. Its incorporation into routine clinical practice may help identify high-risk patients who require more intensive nutritional support and improve their long-term prognosis.

## 1 Introduction

Maintenance Hemodialysis (MHD) serves as a key therapeutic approach for individuals suffering from end-stage renal disease (ESRD) (1), yet these patients often face a high mortality rate and complex complications (2). Malnutrition, specifically protein-energy wasting (PEW), is a prevalent and multifaceted issue among MHD patients, strongly linked to inflammation, atherosclerosis, and a range of unfavorable consequences, such as infections, cardiovascular diseases, and increased hospitalization rates (3–5). Therefore, accurately assessing the nutritional status of MHD patients is crucial for improving their long-term prognosis.

Numerous tools have been developed to assess nutritional risk. The Subjective Global Assessment (SGA) and the Malnutrition-Inflammation Score (MIS) are commonly used but rely on subjective evaluation, requiring trained professionals to ensure reproducibility (6). While the MIS has been shown to be superior to SGA and correlates with clinical outcomes in hemodialysis patients (7), there is a need for simple, objective, and quantitative indicators.

The Geriatric Nutritional Risk Index (GNRI), as an adaptation of the Nutritional Risk Index (NRI), serves as a straightforward and objective tool to evaluate nutritional condition (8). It incorporates objective parameters: body weight, height, and serum albumin concentration. Hypoalbuminemia and low Body Mass Index (BMI) are key components of PEW and are established predictors of mortality in various populations, including dialysis patients (9). The combination of albumin and BMI in the GNRI has been demonstrated to enhance the predictive power for patient outcomes compared to either parameter alone (10).

The prognostic value of GNRI has been extensively studied. A landmark study by Yamada et al. (11) demonstrated that a low GNRI was a strong predictor of mortality in a large cohort of hemodialysis patients. This finding has been corroborated in diverse settings; for instance, GNRI predicts mortality and renal progression in patients with chronic kidney disease (CKD) not yet on dialysis (12), and is associated with cardiovascular events and all-cause mortality in this population (13). Furthermore, its utility extends beyond nephrology, predicting outcomes in patients undergoing percutaneous coronary intervention (14) and in older adult patients with comorbidities (15).

Given the established link between nutrition and survival in MHD patients, and the proven utility of GNRI in related fields, further validation in specific clinical cohorts remains valuable. The primary objective of this research is to assess the significance of GNRI in predicting the overall survival of MHD patients in our single-center cohort by retrospectively analyzing clinical data and survival rates, thereby contributing to the growing body of evidence supporting its clinical application.

## 2 Materials and methods

### 2.1 Research design

**Figure 1:**
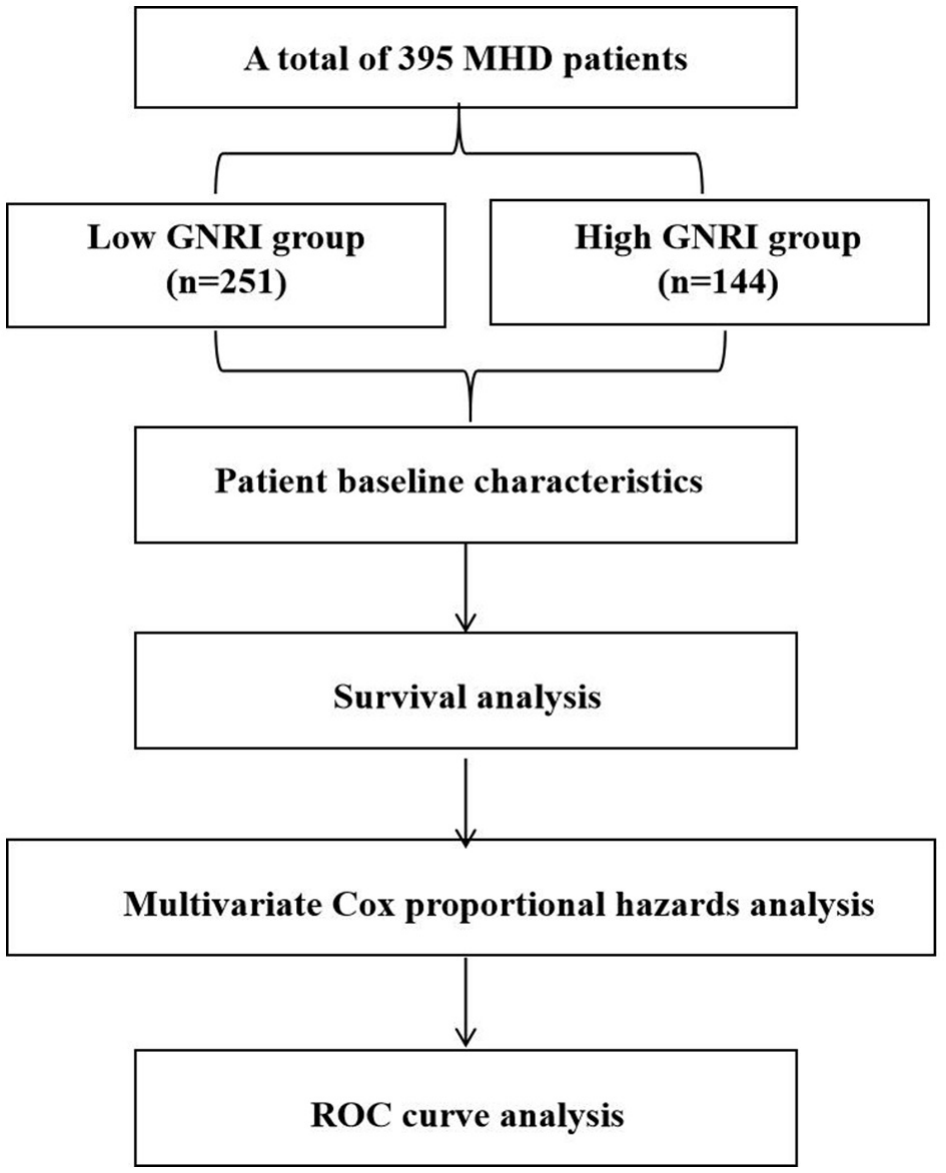
Flow diagram of patient selection for the study, illustrating the inclusion and exclusion criteria applied to the initial patient population, leading to the final analytical cohort of 395 maintenance hemodialysis patients.

A retrospective cohort study was conducted at a single center involving 395 stable long-term outpatient hemodialysis patients from the Nephrology Department of The Second Affiliated Hospital of Wannan Medical College between 2020 and 2024. The endpoint was case death, with the follow-up period ending on December 1, 2024. The study was approved by the Ethics Committee of The Second Affiliated Hospital of Wannan Medical College. Given the retrospective nature of the study and the use of anonymized clinical data, the ethics committee waived the requirement for written informed consent. Detailed information regarding the inclusion and exclusion of patients is presented in Figure 1. Patients were divided into two groups based on their GNRI values: those with GNRI ≤ 93 were assigned to the low GNRI group, while those with GNRI >93 were assigned to the high GNRI group. All patients were followed until the study endpoint (death) or the end of the follow-up period. There were no losses to follow-up, as outcome data (death) was ascertained through mandatory hospital and national registry linkages.

### 2.2 Patient selection

Inclusion criteria:

(1) Hemodialysis duration >6 months before enrollment.
(2) Use of arteriovenous fistula or long-term central venous catheter for dialysis.

Exclusion criteria:

(1) Severe cardiac dysfunction (NYHA Class III–IV).
(2) Malignant tumors.
(3) Recent severe trauma or infection.
(4) Severe obesity or cachexia [Body Mass Index (BMI) >30 kg/m^2^ or < 18 kg/m^2^].
(5) Severe edema.

### 2.3 Sample size consideration

As a retrospective study, all eligible patients from the specified time period were included (*n* = 395). A *post hoc* power analysis was performed using G^*^Power 3.1 software to determine the achieved power of the study. With a total sample size of 395, an observed event (death) rate of 49.87%, a significance level (α) of 0.05, and the obtained hazard ratio (HR) of 0.940 for GNRI from the Cox regression model, the analysis indicated that the study achieved a statistical power of 92.5% for detecting the association between GNRI and overall survival. This exceeds the conventional 80% threshold, indicating that the sample size was sufficient for the primary analysis.

### 2.4 Data extraction

Clinical information was retrieved from the hospital's electronic health records. Patient information including age, gender, and medical history was collected. Fasting measurements of actual height and body weight were taken in the morning. Venous blood samples were drawn to test levels of hemoglobin, albumin, cholesterol, triglycerides, calcium, phosphorus, intact parathyroid hormone, serum creatinine, and cystatin C. Biochemical data from urine and peritoneal dialysis (PD) fluid were combined and used with the software PDA1dquest 2.0 to calculate urea clearance index (Kt/V), creatinine clearance rate (Ccr), and normalized protein catabolic rate (nPCR). Subjective Global Assessment (SGA) was employed to evaluate the nutritional status of patients.

### 2.5 GNRI calculation method

The GNRI is calculated using the method proposed by Bouillanne et al. (16) in 2005 for assessing the nutritional status of older adult hospitalized patients. The formula is:

GNRI = [1.489 × albumin (g/L)] + [41.7 × (actual weight/ideal weight)]. When the actual weight surpasses the ideal weight, the ratio is documented as 1. Conversely, when the actual weight is less than the ideal weight, the actual ratio is recorded. For hospitalized bedridden patients, the ideal weight was calculated using the Lorentz formula (16), based on knee height and age. For general dialysis patients, the actual weight is represented by the dry weight. The ideal weight in this study was calculated based on actual height, assuming a Body Mass Index (BMI) of 22 kg/m^2^ (17).

### 2.6 Statistical analysis

Statistical analyses were conducted using SPSS software version 20.0. Normally distributed continuous data were presented as mean ± standard deviation (SD), with intergroup comparisons performed via analysis of variance (ANOVA). Non-normally distributed data were depicted as medians (range), and group comparisons utilized non-parametric tests. Categorical variables were expressed as percentages, with chi-square tests employed for intergroup comparisons. Kaplan–Meier survival curves were constructed to estimate survival rates, while multivariable Cox proportional hazards models were applied to identify prognostic risk factors. The prognostic predictive value of the observed index was evaluated through ROC curve analysis. To compare the predictive value of GNRI against its component, serum albumin, separate univariable Cox regression models were built. The predictive abilities of GNRI and albumin were compared using the Area Under the Curve (AUC) from receiver operating characteristic (ROC) analysis. The DeLong test was used to compare the AUCs. A *p*-value less than 0.05 was deemed to indicate statistical significance.

## 3 Results

### 3.1 Patient baseline characteristics

This study encompassed a total of 395 participants and were divided into two groups based on the GNRI: the GNRI ≤ 93 group (251 cases) and the GNRI >93 group (144 cases). There were notable disparities between the two groups regarding age, BMI, years on dialysis, hemoglobin, albumin, triglycerides, phosphorus (*p* < 0.05). Specifically, the average age of patients in the GNRI ≤ 93 group was 54.02 ± 16.09 years, which was significantly higher than the 45.61 ± 11.08 years in the GNRI >93 group (*p* < 0.001). The average BMI in the GNRI ≤ 93 group was 18.73 ± 1.26 kg/m^2^, significantly lower than the 21.02 ± 2.48 kg/m^2^ in the GNRI >93 group (*p* < 0.001). The years on dialysis were 67.09 ± 36.12 years in the GNRI ≤ 93 group, significantly more than the 47.89 ± 22.15 years in the GNRI >93 group (*p* < 0.001). In terms of hemoglobin levels, the GNRI ≤ 93 group had 105.42 ± 11.03 g/L, while the GNRI >93 group had 125.09 ± 13.69 g/L, with a statistically significant difference (*p* < 0.001). Albumin levels were 35.39 ± 2.13 g/L in the GNRI ≤ 93 group and 41.41 ± 3.64 g/L in the GNRI >93 group (*p* < 0.01). Triglyceride levels were 1.39 ± 0.72 mmol/L in the GNRI ≤ 93 group and 2.03 ± 1.35 mmol/L in the GNRI >93 group (*p* < 0.001). Additionally, phosphorus levels were 1.42 ± 0.37 mmol/L in the GNRI ≤ 93 group and 1.73 ± 0.57 mmol/L in the GNRI >93 group (*p* < 0.001). In contrast, hsCRP levels were 1.37 ± 0.47 mg/L in the GNRI ≤ 93 group and 1.81 ± 0.41 mg/L in the GNRI >93 group (*p* < 0.001), see Table 1.

**Table 1:** Demographic characteristics and laboratory measurements of the study population, stratified by GNRI group.

**Items**	**All patients (*n* = 395)**	**Low GNRI group (*n* = 251)**	**High GNRI group (*n* = 144)**	***p* Value**
Age (years)	51.32 ± 15.61	54.24 ± 16.09	45.61 ± 11.08	0.001
Sex (male; %)	189 (47.85)	123 (49.00)	66 (45.83)	0.19
Body mass index (kg/m^2^)	19.52 ± 2.31	18.73 ± 1.26	21.02 ± 2.48	0.001
Years on dialysis (years)	60.45 ± 32.67	67.09 ± 36.12	47.89 ± 22.15	0.001
Hemoglobin (g/L)	112.09 ± 79.41	105.42 ± 11.03	125.09 ± 13.69	0.001
Albumin (g/L)	37.48 ± 4.15	35.39 ± 2.13	41.41 ± 3.64	0.01
Cholesterol (mmol/L)	4.79 ± 0.87	4.68 ± 0.84	4.81 ± 0.98	0.1648
Creatinine (μmol/L)	937.84 ± 203.15	932.63 ± 197.42	942.31 ± 212.89	0.6488
Triglycerides (mmol/L)	1.62 ± 1.03	1.39 ± 0.72	2.03 ± 1.35	0.001
Calcium (mmol/L)	2.31 ± 0.21	2.31 ± 0.24	2.29 ± 0.21	0.4051
Phosphorus (mmol/L)	1.52 ± 0.48	1.42 ± 0.37	1.73 ± 0.57	0.001
hsCRP (mg/L)	1.52 ± 0.52	1.37 ± 0.47	1.81 ± 0.41	0.001
Blood urea nitrogen (mmol/L)	24.64 ± 5.63	24.91 ± 5.30	23.96 ± 5.76	0.0976
Uric acid (mmol/L)	438.57 ± 73.47	440.34 ± 75.68	432.79 ± 72.09	0.3322
B2-microglobulin (mg/L)	29.05 ± 6.36	28.74 ± 6.51	29.89 ± 5.98	0.0826
C-reactive protein (mg/dl)	5.62 ± 3.25	5.79 ± 3.46	5.31 ± 3.01	0.1653
Kt/V	1.36 ± 0.34	1.36 ± 0.31	1.37 ± 0.29	0.7523
Urea reduction ratio	76.32 ± 5.89	76.65 ± 5.86	75.98 ± 5.99	0.2786

### 3.2 Survival analysis

**Figure 2:**
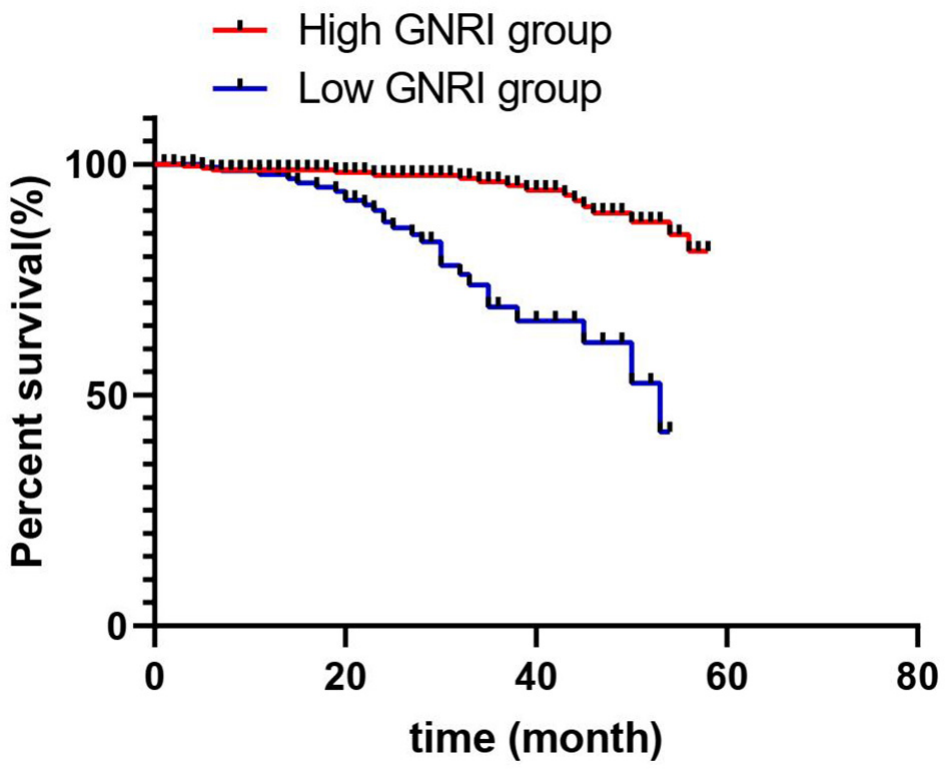
Kaplan–Meier survival curves comparing overall survival between maintenance hemodialysis patients with high GNRI (>93) and low GNRI (≤ 93). The log-rank test was used to assess the statistical significance of the difference between the survival curves.

During the 5-year observation period, out of the 395 patients, 197 died, resulting in an overall survival rate of 49.87%. The Kaplan–Meier survival analysis indicated that the survival rates for the high-GNRI and low-GNRI groups were 68.8 and 25.8%, respectively (*p* = 0.001; Figure 2). The log-rank test revealed that the mortality rate in the high-GNRI group was significantly higher than that in the low-GNRI group (*p* < 0.05).

### 3.3 Comparison of GNRI and albumin

In univariable analysis, both lower GNRI (HR = 0.920, 95% CI: 0.890–0.951, *p* < 0.001) and lower serum albumin (HR = 0.870, 95% CI: 0.825–0.918, *p* < 0.001) were significantly associated with increased mortality. The ROC curve analysis demonstrated that GNRI (AUC = 0.754, 95% CI: 0.708–0.806) had a significantly better predictive ability for mortality compared to serum albumin alone (AUC = 0.698, 95% CI: 0.648–0.748; *p* = 0.018).

### 3.4 Multivariable Cox proportional hazards analysis

The multivariable Cox regression analysis was utilized to assess the impact of various clinical indicators on the mortality risk of patients undergoing maintenance hemodialysis. The model showed good fit, as indicated by a significant likelihood ratio test (*p* < 0.001) and a lower Akaike Information Criterion (AIC = 1,518.2) compared to a null model (AIC = 1,621.7). The results indicated that age is a significant predictor of mortality risk; for each additional year of age, the risk of death increases by 10.1% (HR = 1.101, 95% CI: 1.066–1.138, *p* < 0.001). Furthermore, the Geriatric Nutritional Risk Index (GNRI) demonstrated a significant inverse correlation with the risk of mortality; an increase of one unit in GNRI is associated with a 6.8% reduction in the risk of death (HR = 0.932, 95% CI: 0.901–0.964, *p* < 0.001), suggesting that nutritional status is a key factor affecting patient prognosis (Table 2). Additionally, higher levels of phosphorus (HR = 1.351, 95% CI: 1.012–1.803, *p* = 0.042) and hsCRP (HR = 1.202, 95% CI: 1.021–1.415, *p* = 0.027) were identified as significant independent predictors of increased mortality risk, while higher hemoglobin (HR = 0.978, 95% CI: 0.960–0.996, *p* = 0.018) was associated with a protective effect. Notably, serum albumin and BMI were excluded from the final multivariable model to avoid multicollinearity with GNRI, which incorporates these parameters.

**Table 2:** Multivariable cox regression analysis of mortality risk in maintenance hemodialysis patients.

**Variable**	**HR (95% CI)**	***p* Value**
Age (years)	1.101 (1.066–1.138)	< 0.001
Sex (male)	1.085 (0.662–1.779)	0.748
Dialysis vintage (years)	0.998 (0.991–1.002)	0.405
Hemoglobin (g/L)	0.978 (0.960–0.996)	0.018
Cholesterol (mmol/L)	0.972 (0.715–1.322)	0.851
Creatinine (μmol/L)	0.999 (0.998–1.000)	0.052
Triglycerides (mmol/L)	0.879 (0.562–1.375)	0.576
Calcium (mmol/L)	0.581 (0.268–1.259)	0.170
Phosphorus (mmol/L)	1.351 (1.012–1.803)	0.042
hsCRP (mg/L)	1.202 (1.021–1.415)	0.027
Blood urea nitrogen (mmol/L)	1.008 (0.933–1.089)	0.847
Uric acid (mmol/L)	0.998 (0.994–1.002)	0.712
B2-microglobulin (mg/L)	0.989 (0.966–1.048)	0.3512
C-reactive protein (mg/dl)	0.798 (0.702–0.907)	< 0.001
Kt/V	1.523 (0.651–3.562)	0.731
Urea reduction ratio	1.103 (0.845–2.440)	0.6984
GNRI	0.932 (0.901–0.964)	< 0.001

Body Mass Index and Serum Albumin were excluded from the final multivariate model to avoid multicollinearity with the GNRI, which incorporates these parameters.

HR, hazard ratio; CI, confidence interval.

### 3.5 ROC curve analysis

**Figure 3:**
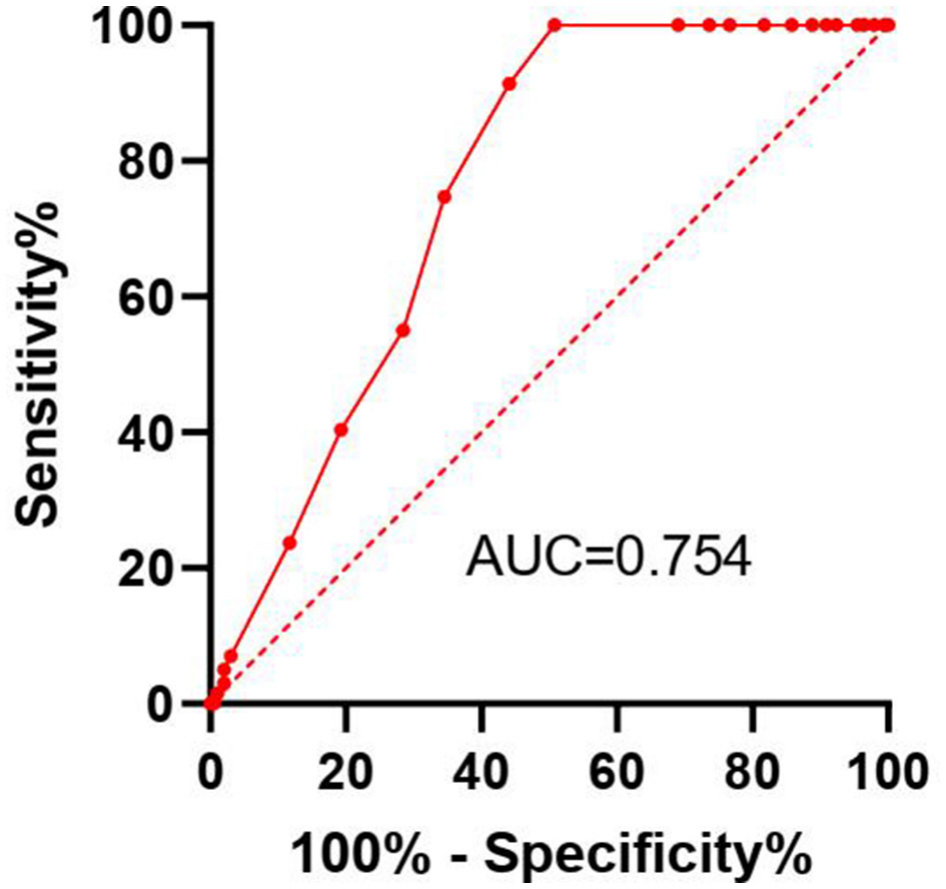
Receiver operating characteristic (ROC) curve for the Geriatric Nutritional Risk Index (GNRI) predicting overall survival in maintenance hemodialysis patients. The Area Under the Curve (AUC) with 95% Confidence Interval is displayed. The optimal cut-off point (93) is indicated.

The ROC curve analysis indicates that the GNRI has a good predictive ability for the overall survival rate of Maintenance Hemodialysis (MHD) patients, with an AUC of 0.754 (95% CI: 0.7085–0.8064; Figure 3). At the optimal cut-off value of 93, the sensitivity was 78.2% and the specificity was 68.5%. This suggests that the GNRI has high sensitivity and specificity and can serve as an effective tool for prognostic assessment.

## 4 Discussion

The present study demonstrates that the GNRI is a valuable predictor of overall survival in patients undergoing maintenance hemodialysis (MHD). Our findings align with previous studies that have established the importance of nutritional status in the prognosis of MHD patients (18). The GNRI, which incorporates easily obtainable clinical parameters such as serum albumin and Body Mass Index (BMI), offers a practical tool for assessing nutritional risk and predicting survival outcomes in this patient population.

Our results indicate that patients with lower GNRI scores have significantly lower overall survival rates, highlighting the impact of malnutrition on mortality in MHD patients. This association may be attributed to the fact that malnutrition can lead to a compromised immune system, increased inflammation, and a higher risk of cardiovascular diseases, all of which are significant contributors to mortality in dialysis patients (19, 20). The inverse relationship between GNRI and mortality risk suggests that addressing nutritional deficiencies could potentially improve patient outcomes.

A study focusing on patients with SCAD undergoing PCI indicated that the GNRIlevel at admission is an independent predictor of all-cause mortality after PCI in SCAD patients (21). Another study analyzed the relationship between GNRI and renal prognosis as well as all-cause mortality in patients with CKD, finding a significant correlation between GNRI and adverse renal outcomes (22, 23). Additionally, research has explored the prognostic value of GNRI in older adult patients with end-stage renal disease (ESRD), highlighting that GNRI is a significant predictor of all-cause and cardiovascular mortality in hemodialysis patients (18).

The ROC curve analysis further supports the predictive accuracy of the GNRI, with an AUC of 0.754, indicating good sensitivity and specificity. This suggests that the GNRI could be used as a reliable prognostic tool in clinical practice to identify patients at higher risk of mortality who might benefit from more intensive nutritional interventions. Regarding the AUC value of 0.754, it is important to contextualize its meaning. While an AUC above 0.8 is often considered excellent, the predictive ability of a single, simple index must be evaluated in the context of the complexity of the outcome. Mortality in MHD patients is a multifactorial event influenced by a wide array of demographic, clinical, biochemical, and comorbidity factors. No single parameter is expected to fully capture this complexity and achieve a near-perfect AUC. In this landscape, an AUC above 0.7 is generally considered acceptable, and above 0.75 is considered good. The value of 0.754 indicates that GNRI has a statistically significant and clinically relevant ability to discriminate between patients who die and those who survive.

Furthermore, the clinical utility of a prognostic tool extends beyond its AUC. The GNRI derives its strength from being an objective, inexpensive, and easily calculable index using routinely collected parameters (albumin and weight). This makes it highly practical for rapid risk stratification in busy clinical settings. As demonstrated by our survival analysis, it effectively identifies a group of patients (GNRI ≤ 93) with a dramatically worse prognosis (25.8% survival vs. 68.8% in the high GNRI group). This ability to categorize patients into distinct risk groups for targeted management is a key application, for which an AUC of 0.754 is sufficient.

The implications of our findings are direct. We propose that the GNRI be incorporated into the standard clinical workflow for MHD patients. Regular calculation of GNRI can serve as an early warning system for nutritional decline and increased mortality risk. Patients identified with a GNRI ≤ 93 should be referred for intensive nutritional counseling, dietary supplementation, and closer follow-up, as part of a multidisciplinary management strategy aimed at reversing protein-energy wasting.

This study has several limitations that should be considered. First, its retrospective nature introduces potential biases, such as selection bias and unmeasured confounders. Second, the data were derived from a single center in China, which may limit the generalizability of our findings to other populations or healthcare settings. Furthermore, as a composite index based solely on serum albumin and body weight, the GNRI does not capture important aspects of nutritional status such as dietary intake, micronutrient deficiencies, or specific nutritional interventions received by patients. The absence of data on protein-energy wasting (PEW) criteria according to established international guidelines (e.g., ISRNM criteria) and detailed dietary habits weakens our ability to establish a definitive mechanistic link between the GNRI and patient survival. In addition, while our multivariate Cox model adjusted for key variables such as age and albumin, it may not have fully accounted for all critical confounding factors. Although measures of dialysis adequacy (Kt/V and urea reduction ratio) were not statistically significant in our model (*p* > 0.05), their inclusion, along with other potential confounders such as specific comorbidities (e.g., diabetes mellitus, cardiovascular disease) and a broader panel of inflammatory markers beyond hsCRP, might have influenced the predictive value of GNRI. Therefore, future multicenter prospective studies that incorporate comprehensive nutritional assessments, including dietary records and PEW criteria, as well as a broader range of covariates, are warranted to validate the predictive value of GNRI in more diverse cohorts and to better elucidate the pathways through which nutritional risk influences outcomes.

## 5 Conclusion

In conclusion, the GNRI is a reliable and highly practical predictor of overall survival in MHD patients and can serve as a useful tool for identifying patients who may benefit from targeted nutritional interventions. Incorporating the GNRI into routine clinical practice could help in the early identification of patients at risk and facilitate timely interventions to improve survival outcomes. Future research should focus on validating the GNRI in diverse populations and exploring its combined use with other nutritional assessment tools to enhance its predictive accuracy and clinical utility.

## Data Availability

The original contributions presented in the study are included in the article/supplementary material, further inquiries can be directed to the corresponding author.
